# Difference in Risk Factors for Breast Cancer by ER Status in an Indigenous African Population

**DOI:** 10.1155/2013/463594

**Published:** 2013-06-25

**Authors:** M. Galukande, H. Wabinga, F. Mirembe, C. Karamagi, A. Asea

**Affiliations:** ^1^Department of Surgery, College of Health Sciences, Makerere University, P.O. Box 7072 Kampala, Uganda; ^2^Department of Pathology, College of Health Sciences, Makerere University, P.O. Box 7072 Kampala, Uganda; ^3^Department of Obstetrics and Gynaecology, College of Health Sciences, Makerere University, P.O. Box 7072 Kampala, Uganda; ^4^Clinical Epidemiology Unit, College of Health Sciences, Makerere University, P.O. Box 7072 Kampala, Uganda; ^5^Morehouse School of Medicine, 720 West view Drive SW Atlanta, GA 30310-1495, USA

## Abstract

*Introduction*. Breast cancer is the commonest cancer among women globally. In Uganda, it is on the rise, projected at a 4.5% annual ASR increase (age standardized incidence rate). The reasons for this steep increase are not fully established. In the recent past, gene profiling in tumor tissues suggests that breast cancers are divided into subtypes dependent on the presence or absence of oestrogen receptor, progesterone, and human epidermal growth factor receptor 2 (HER 2). These subtypes do have distinctive clinical outcomes and perhaps risk factors from past studies. There is paucity of data on hormonal receptor status and the traditionally known risk factors in sub-Saharan Africa. The purpose of this study therefore was to establish the differences between ER status and the traditionally known risk factors for breast cancer in Uganda. *Methods*. An observational analytical hospital, based study, carried out at Makerere University, College of Health Sciences. Formalin fixed and paraffin imbedded sections were prepared for haemotoxylin and eosin (H&E) stains and immunohistochemistry (IHC). Ethical approval was obtained. *Results*. A total of 113 women were recruited. Mean age was 45 years (SD14). There were no significant differences in selected risk factors (setting, age, contraceptive use, parity, breast feeding, or menarche) by ER status although ER negative tumors had significantly higher grade tumors (by a factor of two) compared to ER positive tumors. *Conclusion*. There were no significant differences among risk factors by ER status contrary to what several other studies suggest. The manifestation of breast cancer in Africa warrants further extensive inquiry.

## 1. Introduction

Breast cancer is the commonest cancer among women globally [1]. In Uganda, breast cancer is on the rise, projected at a 4.5% annual increase in ASR (age standardized incident rate) from 2006 [2], therefore currently approximated to be 40/100,000 from 11.7/100,000 in the 1960s. The reasons for this steep increase are not fully established. In the recent past gene profiling in tumor tissues suggests that breast cancers may be divided into subtypes dependent on presence or absence of oestrogen receptor, progesterone receptor, and human epidermal growth factor receptor 2 (HER 2). Luminal A tumors are (ER+, PR+) (ER−, PR+), HER−, and luminal B tumors are (ER+, PR+), HER2+, TNBC (ER−, PR−) HER2−, HER2+/neu (ER−, PR−) HER2+. The basal type is a subtype similar to TNBC with an overlap of 80% of identifying characteristics. These subtypes do have distinctive clinical outcomes from past studies [3–6]. Although reproductive factors have been known for decades to be associated with breast cancer risk, it is unclear to what extent these associations differ across subtypes defined by ER status [7] in sub-Saharan Africa populations.

Literature suggests that the molecular profiles in breast tumors are generally fixed at inception [6]; exposures that influence the risk of developing breast cancer might be related to the tumor molecular profiles that later affect the biology and clinical behaviour of the tumors that arise. This has not been widely evaluated in an African population where the incidence of breast cancer is rapidly increasing and where disparities exist between breast cancers among Caucasian women in industrialized and African women in developing countries.

Some studies indeed suggest that risk factors may vary by molecular subtypes therefore suggesting different etiologic pathways [8–10].

Among the disparities based on tumor biology is overrepresentation of unfavorable subtypes in indigenous African populations [11, 12].

The purpose of this study therefore was to establish the differences between ER status and the traditionally known risk factors for breast cancer.

## 2. Study Site Context

Uganda is a land-locked country straddling the equator in eastern Africa. The country is 241,040 km and currently has a population of 32,709,865 people [13]. With a total fertility rate of 6.7 births per woman-the second highest in the world-Uganda is due to double its population (starting with 2,006 numbers) by 2037 [14]. The capital, Kampala, is a city of 1.4 million people located in the south-central region of the country. Despite achieving 5.8% gross domestic product (GDP) growth rate in 2010, almost one-third of the country still lives in poverty (defined as living on less than US $1.25/day) [13]. A total of 85% of the population live in rural areas, and most of them work in the agriculture sector [15]. Uganda ranks 143 among the 169 countries surveyed for the 2010 Human Development Index [16, 17]. Life expectancy has slowly been increasing to its current level of 53 years, although half of the populations are between the ages of 0 and 14 years. The Ugandan health system is developed with public and private providers; most of the health care is free, and referral for cancer care is mostly to Mulago National referral hospital.

## 3. Methods

### 3.1. Study Design

This was an observational analytical study.

### 3.2. Setting

Two tertiary care institutions: Mulago Hospital, the Makerere University teaching hospital and Ugandan Cancer Institute. The sole public state funded comprehensive cancer treatment center in Uganda.

### 3.3. Study Sample

Women with a confirmed histological diagnosis of invasive breast cancer were recruited prospectively and consecutively during the period of 2011 to 2012.

Women who had insufficient clinical data (inconclusive histopathological reports) were excluded.

### 3.4. Laboratory Methods and Quality Assurance

We used the quality assurance guidelines of the College of American Pathologists (CAP). The laboratory had control tissue which was proven positive for each of the antibodies; a section of the positive control was used at every run of the day, and a negative control was also run.

Paraffin specimens were cut into 4 sections and mounted on positively charged slides. The slides were paraffinized and rehydrated in xylene followed by graded alcohols then washed in Tris-buffered saline. The immunohistochemical (IHC) assays were performed using an immunostainer with antibodies and antigen unmasking. 

Appropriate negative controls for the immunostaining were prepared by omitting the primary antibody step. The results were scored semiquantitatively using Reiner's four point scale based on intensity and percentage of IHC reaction [4], and HER2 staining was evaluated according to manufacturer's instructions. 

Antibodies used were ER (clone SP-I), ASR PR (clone Y85), ASR, and HER2/neu (c-erbB-2), clone CB-11) and were purchased from Cell Marquee Corporation, Rocklin, CA, USA.

Proxies of these molecular subtypes were determined by immunohistochemical stains of oestrogen receptor (ER), progesterone receptor (PR), and human epidermal growth factor receptor (HER) -2/neu proteins. Together the three markers were used to define four tumor subtypes: luminal A (ER= or PR+ and HER-2 neu−), luminal B (ER+ or PR+, HER-2/neu+), HER2/neu+ (ER− & PR−), and triple negative (ER−, PR− and HER2/neu−).

ER/PR scoring system staining of <5% of tumor cell nuclei was considered negative. Both border line and overtly positive stains were considered positive.

Her2/neu− was considered when no staining was observed or membrane staining was <10% of the tumor cells.

Haematoxylin and eosin (H&E) staining was performed first to confirm diagnosis of invasive breast cancer before immunoassaying. The histological type and grade were determined. All the histological slides were received by an experienced consultant pathologist and laboratory technicians, and the tumors were classified according to Nottingham modification of the Scorff Bloom Richardson criteria [18]. Based on histology, tumors were classified into the following groups: invasive ductal carcinoma (NoS), lobular, medullary, papillary, and colloid.

### 3.5. Statistical Analyses

Subjects were selected on the basis of presence or absence of breast cancer. 

Demographic factors and selected variables (reproductive factors) were compared between ER+/ER− using *t*-tests and chi square tests for categorical variables. *P* value was considered significant if <0.05. Data were occasionally missing for some variables, and no further analyses were done for missing data.

### 3.6. Ethical Consideration

 The study was approved by the Institutional Review Boards of Makerere University and the Uganda National Council of Science and Technology (UNCST). All participants provided an informed written consent.

## 4. Results

### 4.1. Tumor Characteristics by ER Status

Table 1 presents the tumor characteristics by ER status; we considered the age equal to or below 50 years and above 50 years. There were no differences between the ER− and the ER+ tumors, though there were more of >50 year olds in the ER− group. For the rural and urban residences, the frequencies were nearly the same for both groups, so was the stage at presentation. Generally put the stage I & II were considered early disease and stage II & IV late or advanced disease.

For tumor grade, the differences were statistically significant (*P* = 0.001) with more of the high grade (III) tumors falling in the ER− category. Histological types were predominantly ductal for both ER subtypes.

Table 2 presents invasive breast cancer ER subtypes and risk factors. Over all there were no statistically significant differences between reproductive factors (parity, AFB, breast feeding, and contraceptive use) and ER+/− tumors and no differences between nonreproductive factors (age, premenopausal/menopausal, setting: rural or urban, and BMI), and all *P* values were >0.05.

## 5. Discussion

We set out to investigate the extent to which risk factors both reproductive and nonreproductive for breast cancer among indigenous Ugandan women differ by ER status. We hypothesized that there were significant differences between the risk factors and ER status. We anticipated that there were differences based on risk factors, due to differences in aetiological or and tumor progression pathways, as previously suggested in other studies [19–21].

We indeed found some clinical differences in characteristics by ER status, supporting the view of heterogeneity and perhaps differences in aetiological pathways but no significant differences were found by risk factors (especially the reproductive ones).

Over all, ER+ cancers are numerically predominant and consistent with other studies [7]. Even though the sample size was small there is some indication that ER+ tumors included a higher percentage of lobular tumors, similar to other studies; however the frequency of poorly differentiated tumors was the same across the ER groups unlike in other studies. This scenario of high grade tumors is consistent though with the overall picture encountered in sub-Saharan Africa where most women present with late stage disease with mostly poorly differentiated tumors [22–26].

The lack of significant differences between ER status and risk factors could be interpreted in two ways: first, that the environmental risk exposure for both ER− and ER+ group is similar, being in a rural or urban setting did not make a difference. Reproductive factors such as contraceptive use, parity and breast feeding were not any different between the two ER groups. There were more women with higher parity ≥7 in the ER− group by a factor of two, and implications of this high parity and ER− status are unclear.

The literature comparing risk factors by ER status reveal inconsistent results, where some demonstrate differences [19, 20] while others do not [21, 27].

The second explanation could be derived from the knowledge of the origins of ER receptor status that all early premalignant breast lesions are strongly ER positive. Mitogen activated protein kinase (MAPK) activation and methylation of ER promotor reversibly transforms ER positive to ER negative breast cancers. The idea is that ER expression is suppressed during tumor progression [28]; therefore the ER status may depend significantly on the stage at diagnosis. The mechanisms for loss of expression include reversible hypermethylation of the ER promotor that downregulates ER expression in invasive breast cancer tumors [29].

The marked difference in characteristics between the ER+ and ER− groups lie in tumor grade, and the ER− group had more high grade tumor compared to ER+ group by a factor of more than two. This is consistent with previous literature and the knowledge that ER− tumors are more aggressive and carry a poorer prognosis. What is also clear from these data is that there were more ER− than ER+ tumors, a state of overrepresentation of ER negative tumors or underrepresentation of ER+ tumors.

The reasons why the ER− group would have more high grade tumors may be a factor of underlying genetic influence (somatic or germline mutations). What we may require moving forward is genetic exploration studies of ER− tumors in the African environment. The alternative view is that ER+ tumors are underrepresented, and this could be because of the population age structure influencing a predominantly premenopausal breast cancer presentation.

Other studies suggest that the activation of the MAPK receptors at the cell surface can downregulate ER expression in a reversible manner [30], raising hope that reversing ER expression may improve therapeutic response of these tumors.

## 6. Limitations of This Study

It is possible that this picture is influenced by relatively small samples, though the findings are consistent with many others in the sub-Saharan Africa; perhaps a multicenter larger study across countries is warranted.

The HER2+ status diagnostics were limited to IHC; FISH was not done. IHC is performed in more clinical laboratories, it is less expensive and less labour intensive than FISH which requires fluorescence microscopes rather than the light microscope used for routine microscopic evaluation by pathologists. The omission of FISH could have led to an underestimation of HER2+ tumors though it would not affect the overall ER− proportions.

## 7. Conclusion

ER negative tumors had significantly higher grade tumors, and no difference in risk factors by ER status was found contrary to what some past studies suggest. Given the disparities seen between African and Caucasian women, a further wider inquiry is warranted for sub-Saharan Africa.

## Figures and Tables

**Table 1:** Tumor characteristics for the ER+/− cancer subtypes among women with invasive breast cancer, an Ugandan 2012 study.

Characteristics	ER positive *n* (%) 53 (47%)	ER negative *n* (%) 60 (53%)	*P*-value
Age			
≤50	37 (70)	33 (55)	
>50	16 (30)	27 (45)	0.106
Setting^†^			
Rural	31 (60)	31 (52)	
Urban	21 (40)	29 (48)	0.399
Stage*			
I & II	11 (21)	11 (19)	
III & IV	41 (79)	46 (81)	0.809
Tumor Grade**			
I	8 (15)	1 (2)	
II	22 (42)	8 (13)	
III	22 (42)	51 (85)	0.001
Histological types			
Ductal	47 (89)	59 (98)	
Others	6 (11)	1 (2)	0.034

^†^1 missing value.

*4 missing values.

**1 missing value.

**Table 2:** Association between selected breast cancer risk factors and invasive breast cancer ER subtypes, an Uganda study 2012.

Characteristics	ER positive *n* (%)	ER negative *n* (%)	*P*-value
Age			
≤50	37 (70)	33 (55)	
>50	16 (30)	27 (45)	0.106
Setting^†^			
Rural	31 (60)	31 (52)	
Urban	21 (40)	29 (48)	0.399
BMI			
1 (normal)	19 (36)	26 (43)	
2 (underweight)	1 (2)	1 (2)	
3	16 (30)	13 (22)	
4	17 (32)	20 (33)	0.750
Parity			
Nulliparous	4 (8)	2 (3.)	
1–3	22 (42)	20 (33)	
4–6	18 (34)	18 (30)	
≥7	9 (17)	20 (33)	0.211
AFB			
0 (<20 years)	23 (50)	29 (55)	
1 (20–24 years)	12 (26)	17 (32)	
2 (25–30 years)	7 (15)	6 (11)	
3 (>30 years)	4 (9)	1 (2)	0.399
Breastfeeding**			
Yes	39 (75)	46 (78)	
No	13 (25)	13 (22)	0.713
Contraceptive use^00^			
Yes	45 (90)	55 (92)	
No	5 (10)	5 (8)	0.762

^†^1 missing value.

**−1 missing values.

^00^3 missing values.

AFB: Age at first birth.

## List of Abbreviations

ASR: Analyte specific reagents
ER: Estrogen receptor
FISH: Fluorescence in situ hybridization
HER: Human epidermal growth factor receptor
IHC: Immunohistochemistry
PR: Progesterone receptor
TNBC: Triple negative breast cancer.
